# Can You Be Too Old To Practice Medicine?

**DOI:** 10.1016/j.shj.2023.100165

**Published:** 2023-03-14

**Authors:** Anthony DeMaria

**Affiliations:** Judy and Jack White Chair in Cardiology, Sulpizio Cardiovascular Center, University of California, San Diego, La Jolla, California, USA

The question of whether there should be a mandatory age of retirement for physicians has been debated for many years. The controversy has again come to the forefront with the New York Times article by our cardiology colleague Sandeep Juahar^1^ titled “How Would You Feel About a 100-Year-Old Doctor?”^1^ In fact, a casual search on Google and even PubMed reveals a substantial number of articles on the topic. Not surprisingly, the issue of mandatory retirement is very complex with rationale both for and against and strong feelings pro and con. For example, actions taken by the AMA Council on Medical Education and Stanford University related to physician age, health status, and competency have been subjected to revision.^2^^,^^3^ Given the gravity and complexity of the concept, it would be impossible to do it justice in an Editor’s Page. Rather, in the following, I will try to delineate some of the most salient factors that impact any consideration of restricting privileges or mandating retirement as physicians age.

- Life expectancy in the United States is increasing and the population is aging. Not only are people living longer but they are exhibiting increased physiological capacity at older age. The standard quip is that 70 is now the new 50.
- Like the general population, physicians are getting older, and they are practicing longer. In the decade preceding 2019, the median age for physicians at retirement increased from 63 to 68.^4^ In 2015, 26% of practicing physicians were aged more than 65 years and the percentage has been increasing.^3^^,^^4^ We are witnessing something akin to the graying of the medical profession.
- The correlation between physiological and chronological age is very variable. This is almost certainly the most important factor governing the subject of mandatory retirement. Some 60-year-olds function as 40-year olds and some as 80-year-olds.
- Advancing age is accompanied in general by a diminution of physical and cognitive capacity. Evidence exists that there is a decrease in mean cognitive ability of about 20% between the ages of 40 and 75 years in the overall population.^2^
- The decrease in physical and cognitive performance seen in the general population also occurs in physicians and similarly varies greatly from individual to individual.^2^^,^^5^ Data indicate that there is a reduction in clinical performance in older physicians. A report of the Council on Medical Education of the AMA indicated that “poorer performance on quality measures such as mortality and length of stay were more apparent for clinicians aged 60 years and more.”^5^ An observational study of hospitalized Medicare patients found that adjusted 30-day mortality rates were 10.8% for physicians aged <40, 11.1% for physicians aged 40-49 years, 11.3% for physicians aged 50-59 years, and 12.1% for physicians aged ≥60 years.^6^
- Although the United States is 1 of only 7 countries with laws banning mandatory retirement, age mandates do exist for several occupations, particularly those with implications for public safety. Pilots, air controllers, some judges, and Federal Bureau of Investigation agents all must retire or alter activities at a stipulated age. A number of countries, notably Japan and Germany, have age requirements for physicians to retire from academic positions.
- Forced retirement would have a major impact on medical manpower; the effect would vary with age. If retirement was mandated for physicians at the same age as pilots, the existing manpower pool would be decreased by approximately 25%.^4^ Moreover, it is estimated that by 2025 the United States may face a shortage of up to 60,000 to 90,000 physicians.^7^ The addition of a mandated reduction of practicing physicians upon an emerging shortage could have catastrophic consequences on the healthcare system.
- Strong laws exist in the United States that would make implementation of mandatory age retirement extremely difficult to impossible. The Age Discrimination in Employment Act (ADEA, 1967) protects certain applicants and employees aged 40 years and more from discrimination on the basis of age in hiring, promotion, discharge, compensation or terms, conditions, or privileges of employment. The ADEA is enforced by the Equal Employment Opportunity Commission. The Age Discrimination Act of 1975 prohibits discrimination on the basis of age in programs and activities receiving federal financial assistance. These laws are largely based upon the fact that physical and cognitive function can vary widely with age. Although there have been exceptions to these laws based upon Bona Fide Occupational Qualification, these are rare exceptions. While the above laws clearly apply for physician employees, they do not apply as directly to doctors who are independent contractors. Here, the application of the law becomes more complicated. Nevertheless, these laws likely eliminate mandatory retirement for physicians as a solution to impaired performance with aging.
- Although mandated medical and neurocognitive testing is a potential approach to protect against age-impaired physicians, this tactic has multiple problems. If testing is applied only to certain age groups, it is not clear that it would satisfy age discrimination laws. In addition, the criteria for adequate/inadequate performance on such tests are undefined. This is particularly true for physicians who usually score 1 to 2 standard deviations above the general population. Importantly, medical and cognitive tests may not predict one’s function clinically. Finally, it is unclear what action should be taken if the testing detects impairment. Experience, temperment, and empathy gained with age may offset any physiological deterioration.
- Confronted with the dilemma of insuring patient safety in the setting of imperfect tactics to evaluate and deal with possible age-related physician deterioration, some institutions have initiated programs of physician testing. Examples of such institutions include LifeBridge Health, the Virginia Health System, Stanford, and Pittsburgh Universities. It is uncertain if any of these programs could provide a boilerplate approach that could be applied widely.
- There are obviously multiple challenges to insuring patient safety as doctors age. Given this uncertain environment, several approaches have been implemented or proposed to both protect patients and validate the skills of competent physicians.^3^
  ○ *Reactive Assessment*: This is the most common current tactic for dealing with possible age-related diminished performance. Detailed evaluation of the practitioner is undertaken when there is suspicion or evidence of errors in care. A major liability of this approach, in addition to possibly being too late to avoid injury, is the reluctance of physicians to report impaired colleagues.^8^
  ○ *Self-Assessment*: Given the dedication of physicians to deliver optimal clinical care, it would seem obvious that self-reporting of perceived deterioration would not only be the best but a requisite tactic to identify decreased competence. However, cognitive dysfunction could obscure recognition of impairment and physicians may fear the consequences of coming forward.^9^ Unfortunately, it does not seem possible to rely only on physician self-assessment.
  ○ *Peer Assessment*: Credentialling for hospital or other privileges routinely requires an assessment by peers. However, this is typically performed by friends or close colleagues and is subject to being superficial, perfunctory, and biased in nature.
  ○ *Recertification*: Most physicians in the United States have some type of Board Certification, and this typically entails periodic recertification. However, older physicians are usually grandfathered in, and testing is often not closely monitored.
  ○ *Age-Mandated Physical and Cognitive Assessment*: Several institutions have already instituted some form of this assessment. However, as indicated above, the criteria for normal are uncertain as is the relevance to clinical practice. However, most importantly, issues regarding age discrimination law create a major problem for widespread implementation.
  ○ *Mandated Retirement*: This would seem to be a nonstarter. First and foremost, it ignores the great variability between physiological and chronological age. It would conflict with current law prohibiting age discrimination. It would remove from practice a large number of competent clinicians at a time of impending physician shortage. All these issues would apply even to the surgical specialties in which manual dexterity and age are most relevant.

It is easy to view the problem of how to deal with potential age-related impairment of clinical competence among physicians as insoluble and an issue to be grappled with by someone else. A completely satisfactory tactic cannot be identified. Existing laws regarding age discrimination are not favorable and we need all the physician manpower we can get. The medical community is unenthusiastic if not overtly opposed, and there does not seem to be significant concern among patients. However, the deterioration of physical and cognitive function with age is undeniable and physicians are no exception. Moreover, evidence exists that such deterioration can result in suboptimal if not erroneous clinical care. Therefore, something must be done to address the issue. The question is who will do it?

I believe it is incumbent upon the medical community to define and implement a program to evaluate the ability of physicians to deliver high-quality clinical care as they age. As the saying goes regarding so many quality-of-care issues, “if we don’t do it, somebody else will, and probably not as well.” The cornerstone of any program should be recognition of the variability between physiological and chronological age. A mandatory retirement age appears to be a nonstarter. Criteria for competence should be based on performance ability and not age. The most reasonable approach currently appears to be mandated periodic physical and cognitive testing. Such testing should be initiated at a relatively young age, perhaps 40-45 years to avoid age discrimination and identify suboptimal practitioners early in their career. Testing should perhaps be initiated at different ages for different specialties. Given the uncertain criteria to establish clinical competence, the results of testing should be considered with broad boundaries as should any resultant actions Such a program would ensure patient safety and also could be viewed as a type of certification to validate the competence of those physicians who are practicing into advanced age. As one of those physicians, I would view such testing with some anxiety, as I did for all Board examinations. However, I would recognize that the examination was in the best interest of my patients, myself, and my profession.

## Funding

The author has no funding to report.

## Disclosure Statement

The author reports no conflict of interest.
